# From Viscount Knutsford

**Published:** 1918-08-03

**Authors:** 

**Affiliations:** London Hospital


### From Viscount Knutsford.

SIR,—I had determined not to be drawn into further correspondence. But I must ask for small space to reply to one statement of Dr. Chapple which is typical of all. He suggested that nurses were sent out by the London Hospital before they had gained their full certificate of training. In confirmation of this he now quotes what I said fourteen years ago of what had happened some years before even that remote date, and happened under very, very exceptional circumstances fully explained at the time. He knows all this, as I told it to him before when he made the same untrue statement. If our nurses are treated so badly, why do they stay? I asked Dr. Chapple this, and he made reply that they were bound to under agreement. Again untrue. Over 100 of the nurses on our private staff could leave to-morrow if the conditions of their service were as unfair as Dr. Chapple's distorted imagination permits him to describe them. If anyone interested in the London Hospital is the least disturbed by any of Dr. Chapple's statements, I shall be glad to correspond with him.—Yours faithfully,

London Hospital, July 30. KNUTSFORD.

